# Midazolam Infusion and Disease Severity Affect the Level of Sedation in Children: A Parametric Time-to-Event Analysis

**DOI:** 10.1007/s11095-021-03113-w

**Published:** 2021-10-18

**Authors:** Parth J. Upadhyay, Nienke J. Vet, Sebastiaan C. Goulooze, Elke H. J. Krekels, Saskia N. de Wildt, Catherijne A. J. Knibbe

**Affiliations:** 1 Gorlaeus Laboratories, Division of Systems Biomedicine and Pharmacology, Leiden Academic Centre for Drug Research (LACDR), Leiden University, PO Box 9502, 2300RA Leiden, The Netherlands; 2 Department of Paediatrics, St Antonius Hospital, Nieuwegein, The Netherlands; 3 Department of Pharmacology & Toxicology, Radboud Institute for Health Sciences, Radboud University Medical Center, Nijmegen, The Netherlands; 4 Department of Clinical Pharmacy, St Antonius Hospital, Nieuwegein, The Netherlands

**Keywords:** critical care, paediatrics, pharmacodynamics

## Abstract

**Aim:**

In critically ill mechanically ventilated children, midazolam is used first line for sedation, however its exact sedative effects have been difficult to quantify. In this analysis, we use parametric time-to-event (PTTE) analysis to quantify the effects of midazolam in critically ill children.

**Methods:**

In the PTTE analysis, data was analyzed from a published study in mechanically ventilated children in which blinded midazolam or placebo infusions were administered during a sedation interruption phase until, based on COMFORT-B and NISS scores, patients became undersedated and unblinded midazolam was restarted. Using NONMEM® v.7.4.3., restart of unblinded midazolam was analysed as event. Patients in the trial were divided into internal and external validation cohorts prior to analysis.

**Results:**

Data contained 138 events from 79 individuals (37 blinded midazolam; 42 blinded placebo). In the PTTE model, the baseline hazard was best described by a constant function. Midazolam reduced the hazard for restart of unblinded midazolam due to undersedation by 51%. In the blinded midazolam group, time to midazolam restart was 26 h versus 58 h in patients with low versus high disease severity upon admission (PRISM II < 10 versus > 21), respectively. For blinded placebo, these times were 14 h and 33 h, respectively. The model performed well in an external validation with 42 individuals.

**Conclusion:**

The PTTE analysis effectively quantified the effect of midazolam in prolonging sedation and also the influence of disease severity on sedation in mechanically ventilated critically ill children, and provides a valuable tool to quantify the effect of sedatives.

Clinical trial number and registry URL: Netherlands Trial Register, Trial NL1913 (NTR2030), date registered 28 September 2009 https://www.trialregister.nl/trial/1913.

**Supplementary Information:**

The online version contains supplementary material available at 10.1007/s11095-021-03113-w.

## Introduction

Sedation of critically ill children during mechanical ventilation in paediatric intensive care units (PICU) ensures patient comfort and reduces the incidence of adverse events like accidental self-extubation. Midazolam is used first line in sedating mechanically ventilated paediatric patients, however quantifying the effect of midazolam in adequately sedating critically ill children proved to be challenging [1, 2].

To date, there has been little progress in the characterisation of midazolam pharmacodynamics in children, where large variability in response has been reported [3]. Instead, expressing sedative efficacy as cumulative mg/kg dosing [3], duration in PICU stay [4], duration of mechanical ventilation [5], number of ventilator free days [6], or proportion of time at target sedation [7] is used which provides a general estimate of sedative efficacy, yet fail to take into account the changing circumstances around the patient in PICU that may contribute to the varying patient level of sedation. Furthermore, current methodologies aimed at linking sedative exposure to the level of sedation have not been successful in the (pediatric) critically ill population, and therefore, more sophisticated methods for characterising sedative efficacy in critically ill children are required.

A pharmacodynamic analysis of sedatives in critically ill children is complicated due to the multitude of patient intrinsic and extrinsic factors that can influence sedation and the removal of patients from studies due to discharge. Time to event (TTE) analyses have become increasingly popular for assessments of sedation efficacy [8]. Rather than analysing absolute sedation scores, TTE analyses only assess the occurrence (or lack thereof) of clinically relevant events (e.g. need for rescue sedatives) over time. This data analysis method allows the assessment of multiple constant (e.g. diagnosis) or time-varying (changing disease severity) covariates, and it can handle censoring and small sample sizes [9].

In this study we therefore aimed to assess the effect of midazolam in maintaining adequate sedation in critically ill pediatric patients using a parametric time to event (PTTE) analysis. For this analysis we used data from the ‘daily sedation interruption in critically ill children’ trial (pDSI trial) [10], a randomised controlled trial, designed to assess the impact of sedation interruption on clinical outcomes in critically ill mechanically ventilated PICU patients. In this study, sedative infusions were discontinued and replaced with blinded infusions of either midazolam or placebo. Blinded infusions were continued until patients were assessed as undersedated, at which point unblinded midazolam was restarted, hereby defined as a clinical event. Studying the duration of blinded infusion offers a unique opportunity to quantify the effects of midazolam compared to placebo. Additionally, we assessed the impact of other clinical factors on duration of blinded infusion, such as age, weight, disease severity, clinical diagnosis and number of failing organs, as well as midazolam plasma concentrations.

## Materials and Methods

### Population and Dataset

The DSI study recruited a total of 129 participants from three different study centres, of whom 8 patients did not receive a trial infusion of either blinded midazolam or blinded placebo [10]. From the remaining 121 patients, 79 patients, all from two of the three study centre, were also enrolled for a pharmacokinetic study, and had midazolam pharmacokinetic (PK) sampling information on which a population pharmacokinetic (pop-PK) model was previously developed [11]. This subset was assessed as the primary analysis cohort for model development, while the remaining 42 patients, all from the third study centre, were used as an external validation cohort. For the primary analysis, all information including information on midazolam dosing [10] and the individual PK parameters [11] were incorporated into the PTTE model. For the external validation cohort, all information was available except individual PK for which population PK was used instead based on individual midazolam dosing information.

**Fig. 1:**
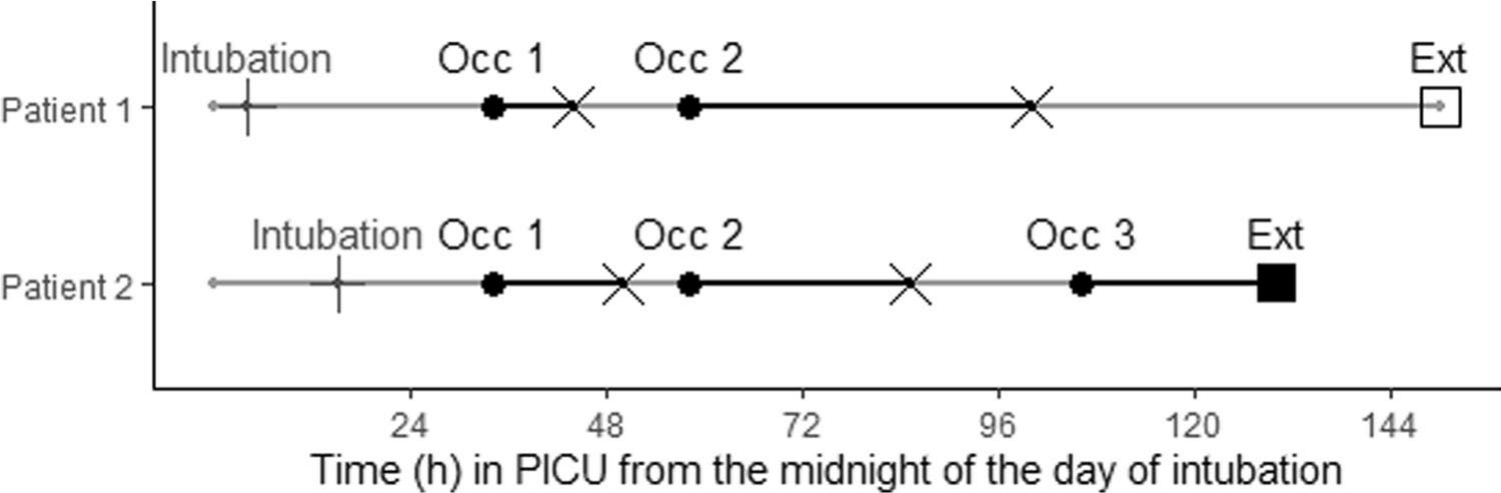
Timeline representation of two hypothetical patients in the DSI study [5], observed from the midnight of the day of intubation (continuous grey line with time of intubation marked as vertical black line). Both patients had multiple occasions of the sedation interruption phase (Occ) during a period of mechanical ventilation (from intubation until extubation [Ext]). The start of the daily sedation interruption phase (start of blinded infusion either midazolam or placebo) was at 10:00 am (black circle). Sedation interruption continued until the patient was too uncomfortable upon which the blinded infusion was ceased corresponding to an event (cross at end of black line). For patient 1, Ext occurred after the end of the second occasion, which was not considered for dropout (open square). For Patient 2, Ext was within the observation period of occasion 3, truncating the assessment of when restart of the infusion would be required if the patient was not extubated. Therefore, time of extubation for patient 2 was considered an event for dropout (black square)

Details on the DSI study are described elsewhere [10, 12] and briefly summarised here. At recruitment in the DSI study, patients were randomly assigned to either the intervention group, which received protocolized sedation with daily interruption by means of a blinded placebo infusion and which will in this work be referred to as the placebo arm (n = 42, for the primary analysis cohort and n = 19 for the validation cohort), or the control group with protocolized sedation only by means of a blinded midazolam infusion and which will in this work be referred to as the midazolam arm (n = 37, for the primary analysis cohort and n = 23 for the validation cohort). Figure 1 illustrates the timeline of multiple occasions in two hypothetical patients from midnight on the day of admission into the PICU. Every morning, a safety screen was conducted on the patients to ensure vitals were acceptable to proceed with sedation interruption. Upon passing the screen, all continuous infusions of sedatives were discontinued and instead, a blinded infusion of either placebo or midazolam was commenced. The start of the blinded infusion was the beginning of the sedation interruption phase. The infusion was continued until a patient was assessed to be undersedated based on a combination of the COMFORT-Behavioural (COMFORT-B) and nurse interpreted sedation score (NISS) (COMFORT-B ≥ 23 or COMFORT-B between 11 and 22, and NISS = 1), which prompted the restart of unblinded midazolam. In patients who received blinded midazolam, unblinded midazolam was restarted at the same dose as prior to the blinded infusion, with an additional bolus dose of midazolam. In the patients receiving blinded placebo, midazolam was started at half the infusion rate the patient received prior to the blinded infusion. At the same time, infusions of morphine and other infusions were also restarted as per the patient regimen. In this analysis, the precise moment at which the blinded infusion was ceased for restart of unblinded midazolam (end of the sedation interruption phase), was defined as an event. Patients were included until extubation.

Patients were screened on a daily basis during their PICU stay, allowing for multiple occasions of sedation interruption. Up to three occasions of sedation interruption per patient were included in the analysis. Subsequent occasions were removed from the PTTE analysis due to the limited number of patients per occasion. In Fig. 1, the end of each occasion of blinded infusion marks an event for the PTTE. Extubation during a blinded infusion (e.g. patient 1 at occasion 3) was assessed for an informative dropout model (see under PTTE analysis below).

### PTTE Analysis

Generally, a PTTE analysis assesses the ‘survival’ of a population over a period of observation pertaining to the loss of individuals in the population due to death, relapse or any other defined event. In the current analysis, an event was described as the commencement of unblinded midazolam at the end of a sedation interruption phase due to undersedation (Fig. 1, black cross). Equation 1 describes for the current study the hazard at time t ($h\left(t\right)$) as a product of the baseline hazard over time (${h}_{0}\left(t\right)),$ the incorporated continuous and categorical covariates (${\lambda }_{Cont}$ and ${\lambda }_{Cat}$, respectively) and a drug effect ($Eff$).

1 $$h\left(t\right)={h}_{0}\left(t\right)\cdot {\lambda }_{Cat}\cdot {\lambda }_{Cont}\cdot Eff.$$

Furthermore, a dropout model was used to account for data missingness in patients who were extubated during an occasion of sedation interruption and therefore censored (dropped out) from the analysis (Fig. 1, patient 2, black box). Dropout is considered informative if there is a relationship between a clinical event hazard and censoring [13]. In this study, dropout as a result of extubation removes patients from the analysis, and therefore potential events which can occur if the patient remains in the study, cannot be captured. Therefore, we simultaneously tested a constant hazard model for informative dropout.

### Baseline Hazard Distribution

The baseline hazard h_0_ of an event occurring at time was evaluated using various constant and time-varying functions such as a step-wise function [14], Gompertz and Weibull functions [15]. Model selection was based on the largest drop in objective function, clinically realistic parameter estimates, and relative standard errors of the estimates < 50%.

### Covariate Analysis for Continuous and Categorical Covariates, and Drug Effect

Covariate analysis was first performed on the clinical event hazard model and then on both the clinical event hazard and dropout model. For the clinical event hazard model, covariates were added in a stepwise forward selection process for a statistical improvement of *p* < 0.05 (dOFV < − 3.84) and eliminated if a selective exclusion from the final model did not worsen the model by significance of *p* > 0.01 (dOFV > 6.63 per parameter excluded). For the dropout model, covariate analysis was conducted by simultaneously incorporating each covariate on the event and dropout event. The covariate was only added to the model in case of a statistical improvement of *p* < 0.05 (dOFV <  − 5.99 for two additional parameters, one each for clinical event and dropout event). Additional selection criteria for covariates included the overall impact of the estimated parameter on the event hazard or dropout event hazard, and relative standard errors of parameter estimates.

The influence of dichotomous categorical covariates (COV) such as reason for admission to PICU, treatment arm (blinded placebo or midazolam), sex, day vs. night, concomitant use of other sedatives and ordinal categorical covariates such as number of failing organs (0–5) and paediatric logistic organ dysfunction (PELOD) scores categorised as 0 (1–3), 1 (10–13), 2 (20–23) and 3 (30–33), were tested as a proportional difference (${\Theta }_{n}$) from baseline hazard of the most frequent category using Eq. 2.

2 $${\lambda }_{Cat}=\left(1+COV\cdot {\Theta }_{n}\right).$$

Continuous covariates such as weight and age were tested as exponential functions. Disease severity scores such as paediatric risk of mortality (PRISM II) and paediatric index of mortality (PIM II), both logistic regression models estimating risk of mortality, were also assessed as continuous covariates. Equation 3 describes the influence (${\lambda }_{Cont}$) of the continuous covariate ($COV$) as an exponential equation with gradient (${\Theta }_{n}$) centred to the median covariate value ($CO\mathrm{V}$ − $CO{V}_{median}$).

3 $${\lambda }_{Cont}={e}^{{\Theta }_{n}\cdot (COV-CO{V}_{median})}$$

The effect of midazolam plasma concentrations (C(t)), cumulative midazolam area under the curve (AUC) from the first administration of midazolam until clinical event, midazolam AUC in the six hours prior to time t, daily midazolam AUC and midazolam AUC over the duration of the sedation interruption phase on clinical event hazard at time t were tested in a proportional or sigmoidal function [15]. Individualised PK parameters used to predict midazolam concentrations over time for each individual patient of the DSI study were obtained from Vet et al. [11].

Equation 4 describes the effect ($Eff$) of midazolam as a proportional difference from the baseline hazard where ${E}_{max}$ is the maximum effect of midazolam on the clinical event hazard, EC50 is the exposure (i.e. concentration or AUC) at which half of ${E}_{max}$ is reached, $\gamma$ identifies the hill-coefficient, and M(t) identifies the exposure of midazolam at time t.

4 $$Eff=1+\frac{{E}_{max}\cdot M(t{)}^{\gamma }}{E{C}_{50}^{\gamma }+M(t{)}^{\gamma }}.$$

Hazard over time from Eq. 1 is then integrated to obtain the model-derived proportion of patients who had yet not experienced the event of restarting unblinded midazolam at time t. (Eq. 5).

5 $$S\left(t\right)={e}^{-{\int }_{0}^{t}h\left(t\right)}.$$

### Model Evaluation

Model evaluation was performed using a 1000-sample bootstrap analysis and a Kaplan–Meier visual predictive check where the median survival curve (Eq. 5) of 200 model-based simulations were overlaid on a Kaplan–Meier curve of observed data of the primary analysis cohort with a 95% confidence interval of the simulations. Furthermore, the final model was also assessed on the observations of the external validation cohort as a Kaplan–Meier curve of observations, with model-based simulations (n = 200) overlaid to assess model fit.

### Softwares and Applications

Data preparation, statistical analysis, graphical representations, and manuscript preparation were conducted on R (v. 3.6.1), using the graphical interface R-studio (v1.2.5019). Model development was performed on NONMEM v 7.4.3 using the Pirana workbench (v. 2.9.9) and the Perl-speaks-NONMEM (PsN v.4.9.0) library of modules.

## Results

A total of 138 events in the 79 patients was available for the primary analysis cohort with all patients having at least one occasion of sedation interruption (blinded midazolam or placebo), 41 patients having a second occasion, and 18 patients having a third occasion. The validation cohort consisted of 75 observations in 42 patients who had at least one occasion, with 21 having a second occasion, and 12 having a third occasion of blinded infusion. Viral respiratory insufficiency and pneumonia were the leading causes for admission to PICU, accounting for 64% of all cases. Patient PRISM II scores ranged from 0 to 44, and the paediatric index of mortality ranged from 0.1 to 45%. Midazolam continuous infusions were administered at a similar rate in both arms prior to the first blinded infusion (median 150 µg·kg^−1^·h^−1^ (IQR 100–224 µg·kg^−1^·h^−1^)). Patient characteristics in both primary analysis and validation cohorts for each arm (placebo and midazolam) are reported in Table 1.

**Table 1:** Summary of patient characteristics in the blinded midazolam and blinded placebo arms for the primary analysis cohort, external validation cohort and the total study

Summary statistics	Primary analysis (Midazolam)	Primary analysis (Placebo)	External validation (Midazolam)	External validation (Placebo)	Total
Age (n [%])					
A. 0–30 days	6 (16)	7 (17)	5 (22)	4 (21)	22 (18)
B. 30 days–2 years	21 (57)	24 (57)	14 (61)	13 (68)	72 (60)
C. 2–18 years	10 (27)	11 (26)	4 (17)	2 (11)	27 (22)
Sex (n [%])					
Male	23 (62)	23 (55)	15 (65)	12 (63)	73 (60)
Female	14 (38)	19 (45)	8 (35)	7 (37)	48 (40)
Diagnosis (n [%])					
Viral respiratory insufficiency	17 (46)	25 (60)	10 (43)	9 (47)	61 (50)
Pneumonia	6 (16)	3 (7)	4 (17)	4 (21)	17 (14)
Other	14 (38)	14 (33)	9 (39)	6 (32)	43 (36)
Patient characteristics (median [lower quartile, upper quartile])					
Weight (kg)	4.60 (3.60, 12.00)	5.69 (3.90, 10.8)	4.40 (3.79, 8.45)	4 (3.50, 5.25)	5.00 (3.70, 10.0)
PRISM II	16 (12, 20)	15 (13, 23)	15 (11, 23.50)	16 (12.5, 24)	16 (12, 22)
PIM 2 (%)	3.16 (2, 7)	5.02 (2.26, 10.3)	1.95 (1.06, 6.48)	1.85 (1.30, 7.35)	3.61 (1.62, 7.80)
PELOD	11 (11, 13)	11 (4, 12.75)	11 (11, 20)	11 (10.50, 17)	11 (11, 20)
Study specific characteristics (median [lower quartile, upper quartile])					
Duration of first intubation (days)	4 (3, 6)	5 (4, 7)	5 (3, 7)	6 (4.50, 7)	5 (4, 7)
Length of stay in P-ICU (days)	8 (6, 14)	7.50 (5.25, 12.50)	7 (5, 15.50)	7 (5.50, 13)	8 (5, 14)
Safety screens					
Passed	4 (3, 5)	3 (2, 4)	3 (2, 5.50)	3 (1, 4)	3 (2, 5)
Failed	0 (0, 2)	0 (0, 1)	0 (0, 0.50)	0 (0, 1)	0 (0, 1)
Total	4 (3, 7)	4 (3, 5)	4 (3, 6)	5 (4, 6)	4 (3, 6)

### PTTE Analysis

A constant baseline hazard well described the hazard of requiring restart of unblinded midazolam over time and was therefore preferred over time-varying hazard functions, Gompertz or Weibull functions that did not result in improved fits.

**Fig. 2:**
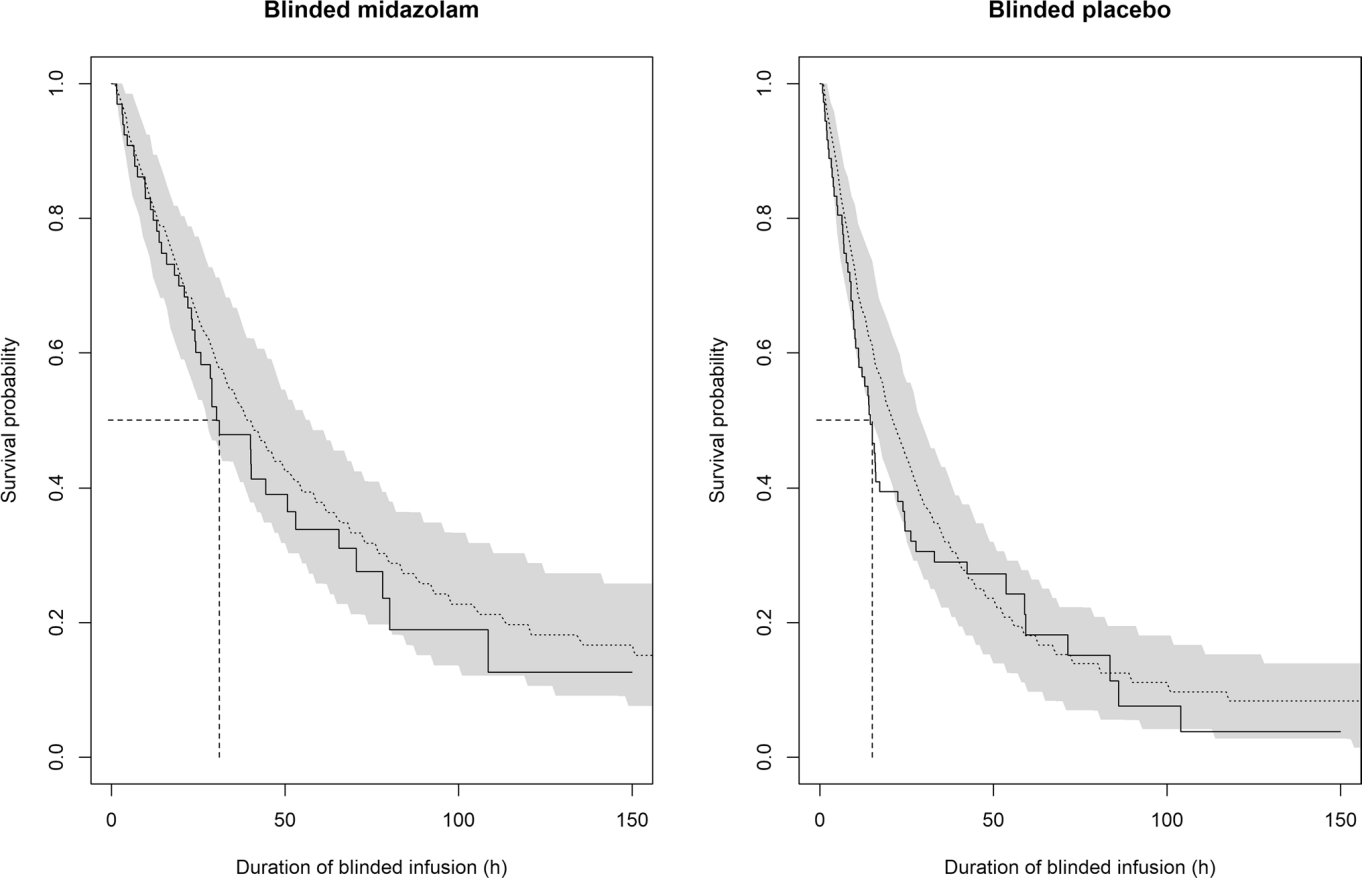
Kaplan–Meier visual predictive check representing the probability of remaining adequately sedated, i.e. surviving without requiring the restart of unblinded midazolam infusion (event) in all three analysed occasions of sedation interruption for patients administered blinded midazolam (left) or blinded placebo (right) as predicted by the final PTTE model. Observed survival for blinded placebo and blinded midazolam (solid black line) are presented with predicted survival (dotted grey line) overlaid on the 95 percent confidence intervals of the predictions (shaded grey area). 50% survival probability of the observed population (dashed line) is marked at 31 h and 15 h, for blinded midazolam arm and blinded placebo arm, respectively(n = 200 simulations)

In the covariate analysis, randomisation to the blinded midazolam treatment arm during sedation interruption resulted in a reduction of 51% in the hazard of requiring a restart of the unblinded midazolam infusion compared to patients randomised to the blinded placebo arm (*p* < 0.01, − 9.47 dOFV). This finding is illustrated in Fig. 2 which is a Kaplan–Meier visual predictive check (VPC) with patients stratified by treatment arm during the sedation interruption phase. The figure shows that the model predicted survival is in agreement with the observed survival of the patient population for both arms of the study, with observed survival falling within the 95% confidence interval of the model with the exception of small trends towards overprediction of the number of patients requiring midazolam restart in the earliest hours. Patients receiving blinded midazolam are observed to have a longer median duration of adequate sedation during the sedation interruption phase compared to blinded placebo with estimated median time to require restart of midazolam at 31 h and 15 h for blinded midazolam and blinded placebo, respectively.

**Fig. 3:**
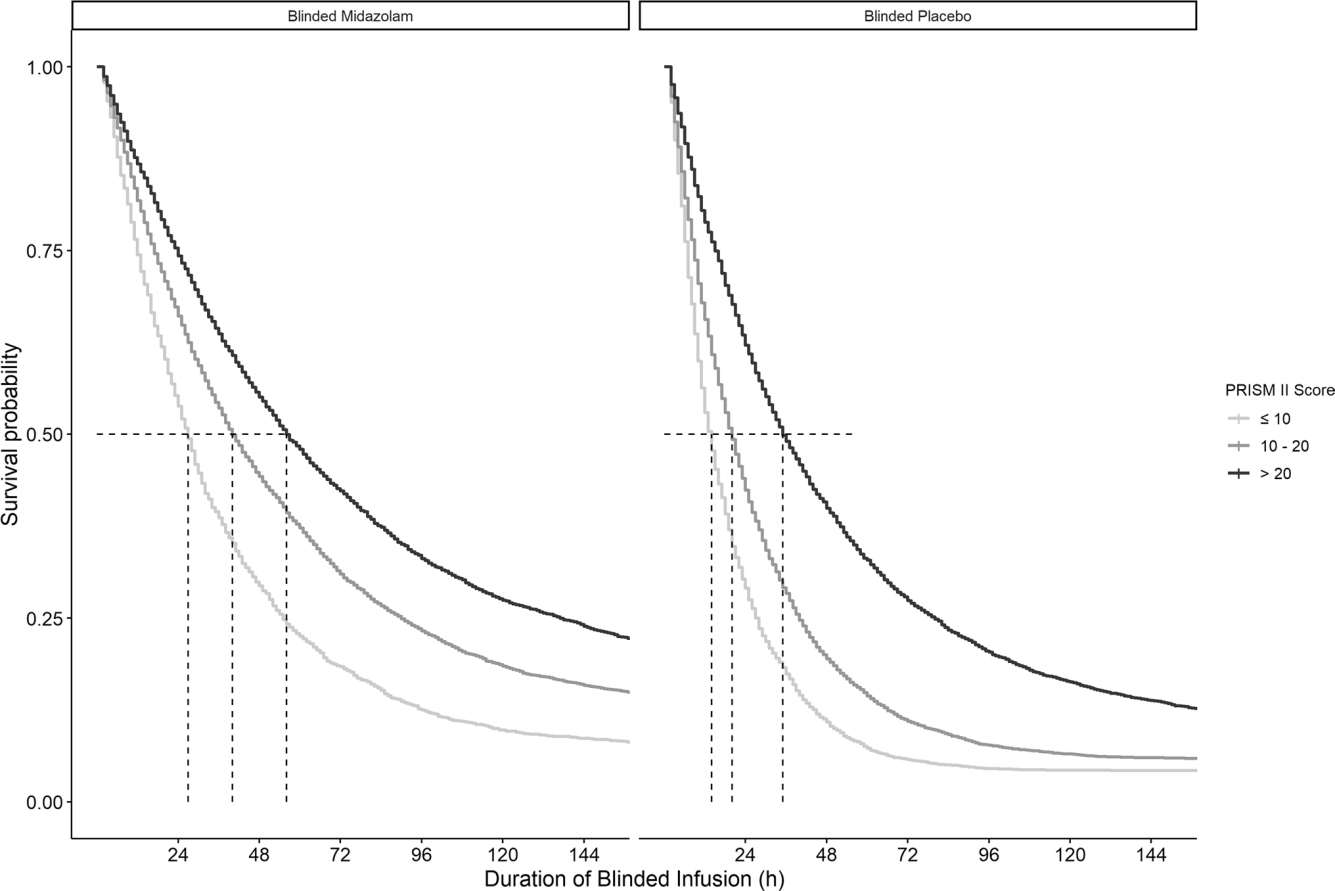
Kaplan–Meier curve representing the increasing probability of remaining adequately sedated, i.e. surviving without requiring a restart of unblinded midazolam, over the duration of blinded infusion (h) as predicted by the model for disease severity categories (PRISM II scores < 10, 10–20, > 20) in patients administered blinded midazolam (left) and blinded placebo (right) (n = 200). Time at 50% probability of requiring a restart depicted as a dotted line for each category

As a second covariate, patient PRISM II scores, incorporated as a continuous covariate into the hazard model reduced the hazard of midazolam restart per unit of PRISM II score increase (*p* < 0.01, dOFV − 9.39), indicating a reducing risk of requiring a restart of midazolam with increasing severity of disease. The negative relationship between PRISM II scores and dosing requirements for midazolam was also reflected in reduced infusion rates of unblinded midazolam administered throughout the study when compared using a Kruskal–Wallis test between the categories of PRISM II scores (*p* < 0.0001, Supplementary Table S2). The relationship of PRISM II scores with the clinical event hazard is illustrated in Fig. 3, median survival for three categories of PRISM II scores (less than 10, 11–20 and above 20) in n = 200 patients are plotted over the duration of blinded infusion. Estimated median time to require a restart of unblinded midazolam was shorter in patients with lower PRISM II scores and ranged from 26 to 58 h in the blinded midazolam arm and from 14 to 33 h in the blinded placebo arm from the lowest to highest category of PRISM II scores, respectively (dashed line).

Other covariates such as those related to midazolam exposure, either quantified by plasma concentration or AUC measures, number of failing organs, reason for admission to PICU, PELOD or PIM II scores, day vs night-time, concomitant administration of other sedatives or other patient specific factors such as sex, age, weight and renal impairment and occasion of sedation interruption were not found to statistically improve the fit of the event hazard. The simultaneous constant hazard dropout model revealed no covariates of influence, and therefore dropout as a result of extubation was deduced to be non-informative.

The parameter estimates of the final PTTE model are reported in Table 2, including the median bootstrap parameter estimates with 90% confidence intervals. All parameters were estimated with relative standard errors below 35% and median bootstrap estimates were within 5% of the model estimated parameters, suggesting the model to be robust and well-supported by the data.

**Table 2:** Parameter estimates and median and 5–95 percentile confidence intervals of a bootstrap (n = 1000 simulations) of the final PTTE model predicting the hazard of the restart of an unblinded midazolam infusion due to undersedation in children receiving blinded midazolam or blinded placebo, reported with % relative standard errors (RSE%)

Parameter	Estimate	RSE%	Bootstrap median	5–95 Percentile confidence interval
Baseline hazard (h^−1^)	0.0373	17	0.038	0.028 to 0.049
$Midazolam$	− 0.506	22.3	− 0.510	− 0.659 to − 0.291
*PRISM II*	− 0.0492	31.9	− 0.051	− 0.078 to − 0.022
Dropout hazard (h^−1^)	0.00237	16.9	0.002	0.002 to 0.003

According to this model, the clinical hazard h for an event (restart of an unblinded midazolam infusion due to undersedation) in children receiving blinded midazolam or blinded placebo is $h(t)=0.037\cdot (1-0.506\cdot Midazolam)\cdot {e}^{-0.049\cdot (PRISM\hspace{0.33em}II-16)}$, which is the product of a constant baseline event hazard distribution, and two covariates, i.e. treatment arm ($Midazolam$) and disease severity ($PRISM\hspace{0.33em}II)$. $Midazolam=1$ for patients administered blinded midazolam and $Midazolam=0$ for patients administered blinded placebo, and $PRISM\hspace{0.33em}II$ is a proportional hazard per unit of disease severity (PRISM II score) centered to a median PRISM II score of 16

For the external validation cohort, the observed events in the Kaplan–Meier VPC (Supplementary Fig. S1) are well described for the placebo arm, as the observed events fall within the 95% confidence interval band on the Kaplan–Meier curve. For the midazolam arm, duration of the sedation interruption phase for 50% of the observed population in the external validation cohort was lower than that of the primary analysis cohort dataset (31 h vs 22 h in patients administered blinded midazolam and 15 h vs 13 h in patients receiving blinded placebo, for the primary analysis and validation cohort, respectively), which was not captured within the 95% confidence interval of the model based simulations (Supplementary Fig. S1).

## Discussion

In this study, we applied PTTE modelling to analyse the efficacy of midazolam in maintaining adequate sedation in critically ill children. To date, few studies have evaluated the pharmacodynamics of midazolam, none being in critically ill children. In this analysis, we utilized data a randomized clinical trial assessing daily sedation interruption in critically ill children that compared blinded midazolam and placebo during the daily sedation interruption phase. The PTTE analysis enabled us to observe a relationship through the estimation of the hazard for experiencing undersedation, in the form of the event of requiring a restart of an unblinded infusion of midazolam after the blinded infusion sedation interruption phase. Two covariates of significance were found to affect time to undersedation in this analysis. The first covariate was the administration of midazolam as part of the blinded infusion, which halved the hazard for requiring a restart of unblinded midazolam and the second, an inverse relationship, between increasing PRISM II scores and event hazard.

Parametric time to event analyses offer an advantageous tool over conventional linear and logistic regression analyses, given their ability to handle the influence of time over the course of the observation period. Time is assessed as part of estimating the baseline hazard for an event through various functions and also in the capacity to handle time varying covariates, such as drug exposure and also clinical pathology results. Furthermore, PTTE analyses also have the capacity to assess the influence of dropout on event hazard, which is of interest in a population prone to dropout during a clinical trial such as the critically ill in PICU.

Using this approach, we showed that patients randomised to (blinded) midazolam had a longer duration of adequate sedation during the interruption phase compared to blinded placebo. In our analysis, the longer durations of blinded infusions in the midazolam arm were reflected as a reduction in the baseline hazard for requiring a restart of unblinded midazolam by 51%. The difference in the duration of blinded infusion between the two arms is distinctly observable in the Kaplan–Meier VPC (Fig. 2) where median time to restart of unblinded midazolam in the observed population is at 31 h vs 15 h in the midazolam arm and placebo arm, respectively.

Although the DSI study highlighted a difference in duration of blinded infusions between the placebo and midazolam arms, a notable outcome of the study was the large proportion of patients on blinded midazolam, who still required the restart of unblinded midazolam, suggesting undersedation is not entirely mitigated by the administration of midazolam. The restart of unblinded midazolam despite infusion of blinded midazolam may lead us to speculate on a lack of efficacy of the applied dosages of midazolam for sedation of critically ill children.

To date, only few studies have linked midazolam concentrations directly to sedation, fewer still in critically ill patients. Studies in adult critically ill patients, have typically correlated midazolam plasma concentrations with increasing ordinal categorical sedation scores, such as the Ramsay sedation scale, albeit with large inter-individual variability [16, 17]. Similarly, another modelling analysis on the influence of midazolam concentration directly on COMFORT-B scores (a composite scoring system for sedation in children of behavioural signs, which was also used in our study) in non-ventilated infants undergoing craniofacial surgery, also identified a relationship between midazolam concentration and sedation, again with large inter-individual variability [18]. Although an influence of midazolam exposure (estimated as plasma concentration and AUC) on the hazard for requiring a restart of unblinded midazolam was not found, the PTTE model was successful in identifying an overall effect of midazolam on prolonging the duration of adequate sedation compared to placebo during the interruption phase, which extends further than previous studies on midazolam in critically ill children [19]. We can speculate the range in midazolam dosages and concentrations in the data of our study is not large enough to identify an influence of midazolam concentration with in the midazolam group.

The hazard for restarting unblinded midazolam was not only influenced by an extrinsic factor (use of midazolam), but also by the intrinsic factor of disease severity, estimated as a PRISM II score upon PICU admission. We were able to conclude on this covariate because in the DSI study, midazolam was infused as per protocolized sedation [10] which limites the potential bias of altering the dose based on disease severity. Higher PRISM II scores related to a reduced hazard for requiring a restart of unblinded midazolam, which resulted in a longer duration of adequate sedation. Figure 3 illustrates an increased survival probability, i.e. longer duration of blinded infusion, for patients with greatest disease severity. An important question for clinicians in this respect is whether patient disease severity at the time of admission needs to be considered when commencing midazolam to reach adequate sedation and avoid overexposure [20].

Our work is predicated on the assumption that COMFORT-B scores accurately quantify sedation, a subjective endpoint, in our population. Theoretically, the decreased midazolam requirements with increased disease severity could be attributed to difference in the performance of the COMFORT-B measurements with increased disease severity. However, it has been shown that this scale can detect treatment-related changes in pain or distress intensity in critically ill children [21]. Moreover, different tools are used to quantify sedation in adults and also in this population. Studies on other sedatives such as propofol in critically ill patients, a similar relationship between disease severity and reduced sedative requirement was reported, with a higher sequential organ failure assessment (SOFA) score being related to an increased sedative effect of propofol [22]. Interestingly, in our study a lower midazolam infusion rate was observed in the highest category of disease severity compared to the healthiest (Supplementary Table S2). This finding further supports that lower midazolam infusion rates may be required in patients with higher disease severity. These findings are applicable to the first days of mechanical ventilation as we only used the first 3 interruption occasions and patients were included within 48 h after becoming eligible (i.e. start of mechanical ventilation).

Some limitations may apply for this study. First, despite the fact that PTTE modelling allows for an investigation into the exposure–response relationship of midazolam, we could not identify an influence of midazolam exposure or concentration on the hazard for restart of unblinded midazolam infusion. For this exploration we had access to the midazolam concentrations and exposure over time in 79 of 121 patients of the DSI study that was published separated using a population PK modelling approach [11]. Instead we only identified midazolam versus placebo as covariate. We hypothesize the range in midazolam concentrations within an individual may have been too small and/or the inter-individual variability in response to midazolam, resulting from large heterogeneity in the small study population, too large. In addition, the presence of a concomitant sedative or analgesic medication in some of the patients could have also played a role in confounding the relationship between midazolam exposure and event hazard. However, as part of the study design, concomitant sedatives and analgesics were also ceased during the sedation interruption phase in patients receiving blinded placebo, and they were continued in the blinded midazolam arm. The influence of concomitant sedatives when tested as a covariate did not further improve the final model for predicting the hazard for requiring a requiring a restart of unblinded midazolam and was therefore not considered to have significantly impact on the analysis. Given the high interindividual variability known to exist in response to midazolam, our study may not be sufficiently powered to estimate an exposure–response relationship. However, with plasma concentration and event data of 79 patients in the primary analysis cohort and data from 42 patients in the external validation cohort, all of which were gathered in a clinical trial on daily sedation interruption, our analysis is one of the largest studies in critically ill mechanically ventilated paediatric patients. In this respect it should be noted that data from the external validation cohort, although obtained in two different centres, were obtained in the context of the same clinical study as the internal data and therefore does not represent a broader clinical population. When more data are available, the current approach with PTTE modelling can be used to further validate our findings. Lastly, to reduce model complexity and maintain identifiability of model parameters, we applied a parsimonious approach in which the impact of the pharmacologically active metabolites was not taken into consideration. We deemed this acceptable as exposure to the midazolam metabolites (AUC) upon i.v. administration has been estimated to be 10–20% of parent exposure [23] and as the pharmacological activity of the metabolites is estimated to be only 50% of the activity of midazolam [24].

In conclusion, the PTTE analysis confirmed a clear effect of midazolam in adequately sedating mechanically-ventilated critically ill children, and also demonstrated a substantial influence of disease severity. By improving our understanding of midazolam requirements, this analysis provides the basis for improved dosing guidelines which in turn will result in a better understanding of the PK–PD relationship of midazolam in critically ill children. The PTTE analysis approach we propose here may also be used to determine the effect of other sedatives and analgesics in vulnerable populations like critically ill children.

## Supplementary Information

Below is the link to the electronic supplementary material.

Supplementary file1 (DOCX 90 kb)
