# Supplementary material for: Midazolam Infusion and Disease Severity Affect the Level of Sedation in Children: A Parametric Time-to-Event Analysis
Source: Pharm Res. 2021 Oct 18;38(10):1711–20. doi: 10.1007/s11095-021-03113-w (PMC8523120; doi:10.1007/s11095-021-03113-w)
Supplement: Supplementary file 1 — Supplementary file1 (DOCX 90 kb) [file 11095_2021_3113_MOESM1_ESM.docx]

**Supplementary Table 1.** Summary of sedation interruption phase specific characteristics in the blinded midazolam and blinded placebo arms for the primary analysis cohort, external validation cohort and the total study.

| Summary Statistics | Primary analysis (Midazolam) | Primary analysis (Placebo) | External Validation (Midazolam) | External Validation (Placebo) | Total |
| --- | --- | --- | --- | --- | --- |
| **Duration of blinded infusion per occasion (h, median [lower quartile, upper quartile])** | | | | | |
| Occasion 1 | 25.73 (11.9, 49.08) | 14.6 (6.41, 34.9) | 25.33 (11.6, 68.2) | 16.9 (8.29, 22.9) | 20.1 (8.03, 44.42) |
| Occasion 2 | 25.0 (10.5, 42.8) | 15.8 (9.42, 25.7) | 17.7 (12.8, 25.1) | 16.2 (7.45, 21.0) | 19.74 (8.80, 28.9) |
| Occasion 3 | 26.02 (19.3, 30.3) | 11.22 (6.50, 15.05) | 11.83 (7.65, 22.2) | 9.00 (6.28, 11.7) | 13.7 (7.97, 27.6) |
| All occasions | 25.8 (12.2, 47.7) | 14.3 (6.85, 33.6) | 21.77 (11.6, 51.0) | 11.50 (7.35, 21.3) | 18.1 (8.25, 40.2) |
| **Duration of blinded infusion per category of PRISM II score (h, median [lower quartile, upper quartile])** | | | | | |
| PRISM II <10 | 23.9 (11.8, 38.4) | 12.1 (9.71, 15.0) | 8.28 (2.46, 16.6) | 10.0 (7.35, 23.5) | 13.3 (7.33, 24.7) |
| PRISM II 10 - 20 | 26.0 (12.8, 42.3) | 9.75 (5.68, 19.8) | 22.2 (11.8, 68.0) | 10.1 (5.75, 20.4) | 16.3 (7.58, 32.4) |
| PRISM II 20 + | 28.1 (12.0, 60.9) | 39.0 (16.3, 57.6) | 24.25 (11.7, 28.9) | 18.4 (11.8, 43.6) | 26.2 (11.6, 53.2) |
| **Rate of midazolam Infusion during unblinded phase (µg·kg^-1^·h^-1^, median [lower quartile, upper quartile])** | | | | | |
| Occasion 1 | 150 (100, 230) | 150 (100, 250) | 150 (100, 200) | 110 (99, 200) | 150 (100, 248) |
| Occasion 2 | 167 (100, 218) | 60.8 (50, 106) | 300 (125, 300) | 100 (52, 113) | 100 (52, 175) |
| Occasion 3 | 151.93 (100, 219) | 200 (100, 241) | 200 (150, 300) | 120 (75, 180) | 154 (100, 234) |
| **Patients per occasion (n[%])** | | | | | |
| Occasion 1 | 37 (56) | 42 (58) | 23 (61) | 19 (51) | 121 (57) |
| Occasion 2 | 20 (30) | 21 (29) | 10 (26) | 11 (30) | 62 (29) |
| Occasion 3 | 9 (14) | 9 (12) | 5 (13) | 7 (19) | 30 (14) |
| **Patients without an event per occasion n(%)** | | | | | |
| Occasion 1 | 12 (18) | 9 (12) | 10 (26) | 4 (11) | 35 (16) |
| Occasion 2 | 6 (9) | 4 (6) | 1 (3) | 3 (8) | 14 (7) |
| Occasion 3 | 4 (6) | 0 (0) | 0 (0) | 0 (0) | 4 (2) |

**Supplementary Table 2.** Median and interquartile ranges for midazolam rates of infusion in patients of increasing disease severity, categorized as PRSIM II scores ≤ 10, 10 – 20 and >20.

| Summary Statistics | n patients (%) | median midazolam infusion rate  (µg·kg^-1^·h^-1^, interquartile range) |
| --- | --- | --- |
| PRISM II ≤ 10 | 12 (15) | 200 (130, 251) |
| PRISM II 10 - 20 | 41 (52) | 149 (100, 216) |
| PRISM II >20 | 26 (33) | 103 (75.0, 200) |

**Supplementary Figure**


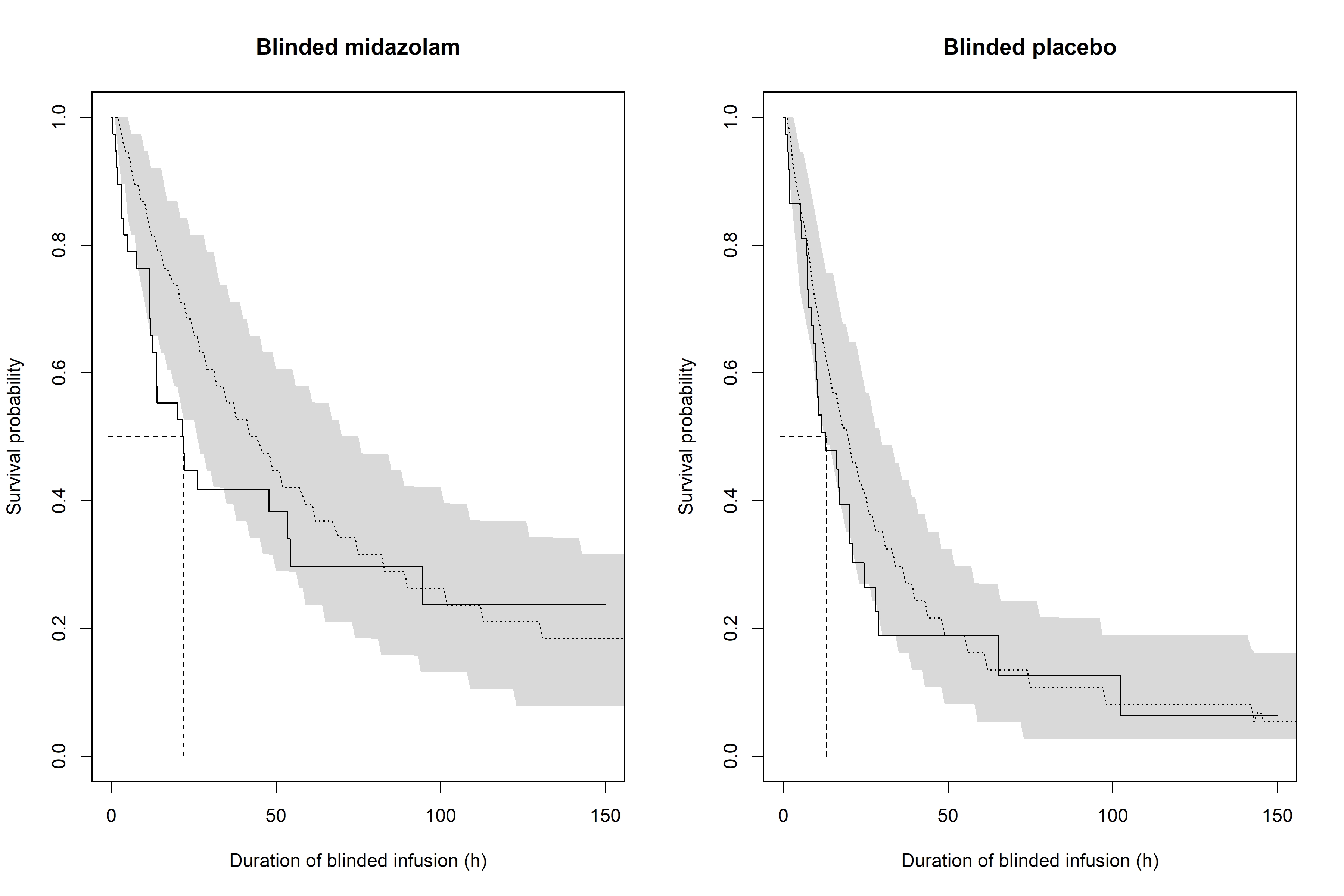


Probability of remaining adequately sedated, i.e. surviving without requiring the restart of unblinded midazolam infusion (event) in all three analysed occasions of sedation interruption for patients administered blinded midazolam (left) or blinded placebo (right) for the validation cohort represented as Kaplan-Meier visual predictive check. Observed survival for blinded placebo and blinded midazolam (solid black line) are presented with predicted survival (dotted grey line) overlaid on the 95 percent confidence intervals of the predictions (shaded grey area) with 50% survival probability of the observed population (dashed line, n=200 simulations).
